# An Anatomical Inquiry Concerning the Nature of Fever

**Published:** 1819-01-01

**Authors:** Robert Wight

**Affiliations:** Edinburgh


### *An Anatomical Inquiry concerning the Nature of Fever.* By ROBERT WIGHT, M. D. Edinburgh.

IN the following Inquiry I propose to myself two questions—1mo. What is fever? 2do. What is the Nature of Fever?

Let us first proceed to the definition of the pyrexiæ, or class of fevers given by Cullen, not inferior perhaps to that of any other Nosologist. "*After shivering, quick pulse, increased heat, lesion of several of the functions, and diminution of strength, especially in the joints.*" These symptoms, be it remembered, are not always present; and if they were, they would not furnish a satisfactory indication of the existence of fever. Any one may have shivering, followed by increase of heat, with concomitant frequency of pulse, whether he labour under fever or not. A person going out in a cold day from a heated room shivers; he walks quickly; his pulse becomes rapid; his skin warm; and next, he sweats profusely. Here are shivering, heat, and sweating, with accelerated circulation, to any extent you please; but no fever. The functions are all *in equilibrio.* When Cullen says, "that there is lesion of several functions," he approaches the truth, but still the idea conveyed is imperfect; for if any part of the body is exempt, it is plain that there is present, not a general affection or disease of the whole body, but that of a part, or a local one; and this cannot be called fever, which spares none of the functions. If, then, we see that all the functions, vital, animal, and natural, are disordered in all cases, however much they may differ from one another, in manner, duration, appearance, and danger; if, in fine, such disturbance of the functions is present in every fever, have we not obtained a certain universal character by which fever is to be distinguished from every other disease? We may, in the mean time, venture to assert, that fever is not to be known by the signs chosen as diagnostic by Dr. Cullen, nor any other of the Nosologists. It is not to be known by frequency of pulse, for the pulse is sometimes slow, sometimes quick; not by increased heat—the heat is sometimes below, and sometimes above that of health; not by shivering, which may occur before, after, or during fever; nor truly can fever either be indicated or defined by any invariable marks, since none such ever existed. As well, let me add, might we attempt to define the ocean by the size of the waves; or the atmosphere, by the shape of the clouds; but if we say, that *fever is disturbance of all the functions,* then we have an infallible indication or comprehensive definition, equally applicable to the whole class, and to all the individuals of every genus, species, and variety of fevers.

Having established the general character of fevers, let us next inquire by what prominent features their various tribes are to be distinguished from one another: this has been a topic of controversy for centuries. The art of medicine, however, is advanced by science, not by eloquence. Diseases are removed by remedies, not by words. That inestimable science and those remedies, are to be found by well devised experiments; by careful observation; by skilful dissection; but not by ingenious hypothesis, or artful disputation.

Cullen referred all fevers to two divisions, the intermittent and continued; the former comprehending the various remittents—and the latter synocha, typhus, and synochus. He has not, however, ascribed any symptoms to synocha, typhus, or to synochus, which are not to be found in the intermittents. Synocha is a fever occurring in the phlogistic diathesis, and is commonly, with sufficient propriety, called inflammatory. Typhus is a fever long distinguished in medical writings by the epithet *putrid;* and occurs in the putrescent diathesis. Synochus, as far as it can be comprehended from the examples given of it, is a chronic fever, or one of long duration, in which the symptoms are sometimes mild, sometimes severe, very variable, and always insidious. Compare all these with the symptoms of intermittent fever, still you find nothing peculiar to the continued. Both intermittent and continued occur in any diathesis, and both may be of long duration, very variable, and very insidious; they are both convertible into each other. The intermittent often becomes continued, and the continued intermittent; therefore, fevers differ in their general properties far less than most medical men are inclined to think. After the strictest comparison, you find only one radical difference among them, and that resulting from the diathesis; and on this evidently depends the species of fever, whether inflammatory or typhoid. Let us add, that every one in the least acquainted with the effects produced on the living body, must acknowledge, that the same causes produce at one time synocha or inflammatory fever; at another time, typhus or putrid, precisely according to the diathesis.

It now seems incumbent on us to consider a little more minutely the nature of each diathesis. The food, liquid and solid, by which the body is nourished, is so changed by the operations of the functions, as to be subservient to the various purposes of life and health, while those changes are properly performed. If the chyme, the chyle, the lymph, the blood, undergo their appropriate conversions; if the nervous energy is suitably regenerated and diffused, all the secretions and excretions are duly formed, and man enjoys the best health—a sound mind in a sound body. This happy condition, when exposed to the influence of the causes of disease, is prone to inflammations and inflammatory fevers, and constitutes the true *phlogistic diathesis.*

But when all things are suffering deterioration; when the chyme, the chyle, the blood, the nervous power, with all the secretions and excretions, are vitiated; when the depraved functions are incapable of any good office; then, upon the application of the causes of disease, that condition of body is prone to putrid affections, to malignant ulcers, and to typhus fevers. This constitutes the putrid or *non phlogistic diathesis.*

Accordingly, there are two things on which fevers necessarily depend, viz. the diathesis and the causes; the diathesis being given, we know of what kind the fever is. Thus, two divisions are spontaneously formed; one comprising the inflammatory fevers, the other the typhoid; and each corresponding precisely with the general definition. Therefore, disturbance of all the functions occurring in the phlogistic diathesis, is inflammatory fever or synocha; but such disturbance occurring in the putrid or non-phlogistic diathesis, is putrid fever or typhus. In this manner fevers, and all their varieties, however complex, irregular, and dangerous they may be, show, at once, to what division they belong. These varieties, in effect, depend on so many circumstances promiscuously combined, as to defy calculation. Fevers differ from each other, according to sex, age, manner of life, habit, climate, soil, season, strength of the causes, and finally, according to the part of the body first attacked, or most severely affected, whether it is external or internal; concerning all of which the physician should most diligently inquire, before he venture to prescribe for the patient.

Having showed by what relationship, and common bond of properties, fevers are connected to one another, and how these are confirmed by a careful comparison of all the symptoms, we are prepared to inquire, whether the signs of fevers really denote any changes to be going on within the body; for if they do denote, them and the signs in number, combination, degree, and succession, correspond with certain corporeal changes, then we have obtained some certain knowledge of fevers, and a fixed principle, according to which they are to be explained.

Dr. Sanders gives the views, and demonstrates the facts which I have brought forward in this Dissertation; and it was during my attendance on his lectures, that the subject first drew my attention. But I did not rest satisfied till I had, by careful observations and dissections, made by myself, found them confirmed in every particular.

Ever since medicine assumed the form of a science, all men who thought profoundly on the means of its advancement, have inculcated the necessity of exploring the bodies of the dead, in order to establish the art on a solid basis; but for many obvious reasons, the progress has been very slow: nor was it before the fall of Constantinople, when literature and science were revived in the West of Europe, that any thing of great utility in this department was accomplished. Then, certainly, anatomists acquired well-merited reputation, by their researches concerning the structure of the different parts of the body when sound, as well as the changes to which they were subjected, during the progress of disease. But not one of them showed, by the aid of the scalpel, in what manner any *function* is disturbed, when under the operation of the causes of disease; nor by what process the organization adapts itself to the new action which has been induced. But if reason, or the fitness of things, ever taught investigation, surely there is nothing more clear, than that the action, and the agent or body acting, must undergo simultaneous change, and of consequence, it should be in our power to discover by dissection, how the part acting has been affected by any morbid action; particularly when that action has been such as to first embarrass the functions, and then destroy life. But laying reasoning aside for the present, let us proceed to the facts.

Anatomists have come to very various and unsatisfactory results, from their examinations of the bodies of those who have died of fever. In one person, certain changes are observed to have occurred in the head; but in another, not. In one, certain changes are observed in the thorax, or in the abdomen; in another not. And finally, cases are recorded, in which no part of the body, external or internal, exhibited any morbid appearances, that is to say, *changes of condition or structure,* to account for the fatal event. Hence, it has been inferred, that anatomy was of no avail, in enabling us to understand the nature of fevers.

Such expositions as those above referred to, never could elucidate fever; for they show nothing more than those remote effects of disease in particular organs, which might occur separately or in combination, without any fever having existed. It is well known, that many perish who have no fever; in whose bodies destruction enough might appear to gratify common curiosity, without leading to the explanation of any thing. Details of such dissections are numberless, but so far as pathology is concerned, they furnish only ponderous monuments of abortive labour. This every diligent student has experienced. Why this unproductive toil? The cause is, that the organization and its functions were not viewed in connection; it was never sufficiently adverted to, that there cannot be any change of structure, unless there have been change of function; that there can be no change of function, unless there have been change in the co-operation of those vital instruments which primarily govern all the movements of the living mechanism, that is, in the relations subsisting between the vascular and nervous systems; that while these co-operate, life continues; when they fail, life languishes; when they cease to co-operate, life ceases; and that all this may happen, as in fevers, though the internal composition of every organ remains unaltered; hence, it is manifest, that in fevers, though the organs are not necessarily changed in structure, yet that their actions or functions are necessarily changed. On what, I ask, depend the actions of all the organs? Doubtless on their nerves.*—Whence do the great majority of their nerves proceed? Certainly from the spinal marrow, within the osseous canal of the spine. Thus, as it were, by the very hand of Nature, are we conducted through the mazes and intricacies of structure, with the utmost certainty, to the source of the greater part of that information which we are anxious to obtain; and we wonder that we were so long bewildered in devious windings, while the highway was plain and open to the very seat of fevers. No process of thought, however, could enable us conceive what changes induced fevers or any other morbid actions. We had opened the spinal canal, and seen the state of the spinal marrow, its membranes, nerves, and their envelopes, before we began to reason on the subject. If you lay open the spine of any one who has died of fever, there you will see what effects the disease has been able to produce throughout the whole canal. All the membranes of the spinal marrow are every where coloured, in consequence of their vessels having been dis-

---

* When nerves are mentioned in relation to functions, their vessels are meant to be included as an active constituent part of them; and so when vessels are mentioned, their nerves are included.

tended with blood; and the same appearances proceed upwards along the spinal marrow, the corpora quadragemina, and base of the brain.* After the dura matral covering is removed, we see all the nerves, where they commence or draw their origins from the spinal marrow, coloured with turgid vessels, in the same manner as the general envelopes themselves. These, I say, are invariable results in fevers, and will not be found inadequate to the illustration of their nature.

The appearances on dissection differ according to the kind and degree of the disorder, and also according to the part and organ which has been most remarkably affected. If it was the synocha or inflammatory fever, both the general envelopes of the *medulla spinalis,* and the visible origins of the nerves are of a lively red; but if it was the typhus or contagious fever, both the general envelopes and the origins of the nerves are of a dark brown; and the same rule holds within the cavity of the cranium. So exactly, I affirm, is the correspondence between the symptoms and the vascular turgescences, that if, during the malady, whether inflammatory or typhoid, the function of any organ whatever was most severely deranged, the visible origins of the nerves proceeding to that organ evince the peculiar effects in the greatest degree. We have, therefore, a host of anatomical facts, proving radically and irreversibly what, in fever, are the manner and kind of change in the relations which ought to subsist between the vascular and nervous systems, at the very centre, where their conjoint action and reciprocal influence preside over all the varying phenomena of animation; at the same time leaving no doubt that this change is the first recognizable link in the chain or series of disorder; and that, by this disorder of functions, if not arrested in its progress, life is ultimately extinguished. In short, let us examine the complete evidence, in all its bearings, and we shall find the whole to unite in this conclusion, that the causes of fever first disturb the circulation of the blood on the ori-

* Though the spine is here chiefly insisted on, there is no intention to convey the idea, that the contents of the cranium are not involved in fevers; the fact is, that the brain and its membranes, in regard to their circulating vessels, always suffer; and during febrile action, the organization there, as in any other part of the body, is liable to injury of every kind; but, if the disease is confined within the skull, *fever does not exist,* nor are the symptoms of it produced. In short, whatever is the condition of the head, *unless the spinal marrow and its appurtenances have been involved, as described in the context, fever never occurs.*

gins of the nerves; that the nerves so affected disturb all the functions, and that hence arise, and thus proceed all fevers.* Now do not the same facts compose that solid basis on which is to be erected the only rational theory, and the only successful treatment of fevers?

*Buccleugh Place, Edinburgh,*

*October*, 1818

---

Dr. Wight thus terminates his Inquiry, after having given, to the two questions proposed on setting out, such answers as the facts seem to him fully to justify. And, truly, if these propositions are solved on sound principles, without inadvertency or fallacy in the reasoning, a very important advancement has been achieved in our knowledge of fever. The two questions, it will be remembered, are—*What is Fever? and, What is the Nature of Fever?*—After showing the inadequacy and flimsy foundation of the former notions and definitions, he analyses the symptoms of fever under its various forms, and finds synthetically, that, as known by symptoms, *fever is disorder of all the functions.*

What follows is of still more deep research and difficulty. To discover the relation which subsists between what is evident and what is occult; to learn how and why symptoms are excited and evolved; to trace in the bodies of the dead certain distinctive and indelible characters inseparable from certain symptoms; to compare the living and the dead in different conditions, states, and degrees; and hence to demonstrate the commencement, progress, and termination of morbid action, and of morbid change in their necessary series and connection, so as to establish by clear induction, the precise nature of disease, requires much labour, circumspection, and accuracy. This has been attempted:—with what success our readers will judge. The very attempt is meritorious. But we are much deceived, if approbation ought to be confined within so narrow limits.

The result of this part of the Inquiry is, that a certain change of action is produced in the centre of the nervous system, in consequence of which the vessels of the membranes of the brain and spinal marrow, as well as those on

---

* Dr. Sanders does not include hectic fever, or that which accompanies phthisical affections; and which he proves to differ anatomically, as well as in symptoms, from those fevers called synocha, synochus, and typhus.

the origin of the nerves, become unusually distended with blood, and that this is the necessary antecedent and concomitant of all the phenomena of fevers.

Admitting, however, that such a state of the vessels of the membranes and nerves of the brain and spinal marrow is anatomically proved to have existed, still it may be questioned, whether it is not the production rather than the precursor of febrile action. But we understand, that Dr. Sanders brings forward an immense accumulation of facts ascertained in diseases both general and local, by which, not only he himself, but those also, who, like Dr. Wight, have heard his lectures, and seen his anatomical demonstrations, are satisfied that, variously modified, a similar change in the relation between the nervous and vascular systems, necessarily precedes and accompanies every disorder of function, whether of one organ, of several organs, or of all the organs of the body. Further, according to him, (Dr. S.) all the truths to be deduced from the states of health and disease, furnish irrefragable testimony, that disorder of function necessarily precedes and accompanies every morbid change in the organization:—so that, if the disorder in the function of any organ were corrected, no such thing could occur as morbid change of structure in that organ; and, with regard to the method of accomplishing this, which is the final cause of the healing art, he affirms, that disorder of function is solely to be corrected by restoring the due relation between the vessels and nerves of the organ disordered in function. This doctrine is not delivered merely in the way of rational conjecture: he has acted on it many years, and the results of his practice afford ample and lucid confirmation. Such are the anticipations which he has formed from the cultivation of this department, that he is accustomed to say,—"When the nervous system and the means of regulating its movements are better known, we shall not, as at present, in instances of tumours and local inflammations, external or internal, be under the necessity of draining the body of its blood, and of using other powerful evacuations, often to the irreparable injury of health. We shall then be able, by the correcting of the action of a few nervous filaments, at once to put an end to the most alarming commotion; as a watch is controlled by the slightest touching of its spring, so will be the living body, the most delicate and exquisite of all constructions, the very contemplation of which inspires the soul with divine enthusiasm."

We perceive then, that this inquiry on fevers, is only a partial sketch of widely extended views of the animal economy, founded on the comparison of the phenomena of life, with the modifications which dissection shows the structure to have undergone. From all the facts thus concentrated, Dr. Sanders infers, that the animate body is liable to no more than two generic diseases, the one *primary*, the other *secondary*. The primary disease is *disorder of function*. The secondary disease is *change in the organic structure*. The order of their occurrence is never inverted. The primary disease may arise and disappear spontaneously, or by the aid of art; but it is impossible for the latter to occur unpreceded by the former. The disorders of function admit of variety, *ad infinitum*. So do the modes of organic change. Still all this is only diversity. Bring together all the lists of diseases, general and local, with their complicated, pedantic, whimsical, and barbarous vocabulary, you will, in every instance, discover either disorder of function, or change of organization, or both combined, nothing more. You cannot discover one example, not even a spot on the surface, or a wart on the finger, unpreceded by disorder of function.

In this manner are subverted all our nosological arrangements, their classes, orders, and multifarious subdivisions, in which diseases, general and local, are fancifully blended and fancifully disjoined; in which inflamed eyes, bleeding at the nose, tooth-ache, and locked jaw, are among *diseases of the whole body*; while the loss of smell, sight, and taste are among local diseases! wherein diarrhœa and menorrhagia are general, and amenorrhœa and constipation, local diseases; wherein simple disorder of stomach and dreaming are diseases of the whole body, while pica and bulimia syncopalis (in which there is inveterate distress of the brain, heart, stomach and bowels, with wasting of the whole body) are only local diseases! Where deficiency of power, or the paralytic state of a *part* is *nervous* disease of the *whole*; while fever (in which *all the nervous system* is involved, often exhibiting in its progress every variety of spasm, convulsion and paralysis) is excluded from among nervous diseases! But the source of this confusion is now manifest: every disease, general or local, is primarily disorder of function, or, in other words, a *nervous disease!*

On this principle Dr. Sanders founds his arrangement of diseases; and shows that, according to it, all morbid affections, of whatever aspect or denomination, find each its proper place without constraint or compulsion.

We expect to be able to lay before our readers his views of the structure, connection, and functions of the contents of the head and spine; of the relations subsisting between the nervous and vascular systems; of the manner in which all those powers which act on the living body so as to sustain life, and maintain or disturb health, influence the economy, and produce their peculiar effects.

Meanwhile we may notice his idea of the intricate doctrine of sympathy, and of those affections termed *sympathetic* and *symptomatic*. He maintains, that sympathies exhibit nothing different in nature, from the common phenomena of vitality. The two eyes co-operating or acting in succession, or both becoming diseased from an impulse on one of them, is nothing different in nature, from the actions of the superior and inferior extremities; from those of both nostrils; of both sides of the tongue; of both sides of the heart; of both sides, in short, of the body or of its contents; all of which may have increased or diminished actions, separately, conjointly, or in succession. Nor are these actions different in nature, from the disturbed action of the stomach when connected with disorders of the heart, the kidnies, any internal organ, or even with that of the skin. Disjunct and unequal actions have got the distinctive appellation of sympathy, while *united and equal actions, (which in reality constitute perfect sympathy)* are not even considered as the same in kind. The former are pronounced miraculous and inscrutable, while the latter are believed to be the ordinary occurrences easily understood. But he endeavoured to prove, and some think, not in vain, that both are equally misunderstood; and, that both depend on the same principle modified, in effect, by the state of the constitution and the causes conjointly. He further endeavours to demonstrate what that principle is.—"Take for example," says he, "those fevers called symptomatic, in which it is confusedly meant, that the symptoms do not indicate real fever, because they depend on some other disease, some local affection in the system. Can all the phenomena be present where fever itself has no existence? Can the signs arise without the thing signified? If so, the definition above given would be a combination of vain words; all the functions might be disordered when there was no fever! and, therefore, fever must continue to be like the subtile ether of philosophers, a vague, indefinable, inscrutable something. But let us examine all the causes, and all the precursor and concomitant indications, and we shall find that there is never such a thing as a cause or causes at once applied to the *whole body* in the production of any fever; in every instance *some part* is first attacked, and that part involves the whole system. Hence, another practical maxim: discover the part and correct its aberration; and you preserve the body from general disorder, that is, fever.

Where now is the difference in nature, between those fevers called sympathetic, and the others called idiopathic, by which is meant fever *per se*, or not depending on any local affection. When a child was inoculated for the small-pox, was the fever which ensued not a real fever? demanding imperiously all the necessary precaution and treatment? Or was the general commotion excited, only symptomatic of the scratch in the arm? Was the disease produced by accidental contagion, different in nature from that produced by inoculation? How does it happen, that when fevers are prevalent, some have headaches; some sore eyes; some affections of the throat; some of the thorax or abdomen; some of the loins, and some of the extremities; which have been removed without fever supervening: while others, who had at first the same local ailments, were gradually involved in complete fever? Compare, critically, all the fevers which rage epidemically, and are distinguished by the epithet *idiopathic*, with the *symptomatic*, or those which supervene exclusively, as is imagined, on local affections, and they all warrant one inference, that an offending part, if not counteracted, brings the whole system, every function into disorder. Nature knows no such distinction as fevers, *idiopathic* and *symptomatic*.

This consideration is connected with a most interesting subject, which Dr. S. has long prosecuted, that is, the *manner in which any one organ or part, when diseased, either in function or structure, involves the rest of the body in disorder*. But we must here conclude for the present, as we only intended to illustrate the scope of Dr. Wight's inquiry; to give some idea of the extent and object of the investigation to which it refers; and, finally, to furnish our readers with topics of reflection, preparatory to a full and detailed discussion.

